# Galectin-3 supports stemness in ovarian cancer stem cells by activation of the Notch1 intracellular domain

**DOI:** 10.18632/oncotarget.11920

**Published:** 2016-09-09

**Authors:** Hyeok Gu Kang, Da-Hyun Kim, Seok-Jun Kim, Yunhee Cho, Junghyun Jung, Wonhee Jang, Kyung-Hee Chun

**Affiliations:** ^1^ Department of Biochemistry and Molecular Biology, Yonsei University College of Medicine, Seoul, Republic of Korea; ^2^ Brain Korea 21 PLUS Project for Medical Science, Yonsei University, Seoul, Republic of Korea; ^3^ Department of Life Science, Dongguk University, Seoul, Republic of Korea

**Keywords:** galectin-3, cancer stem cells, Notch1, ovarian cancer

## Abstract

Ovarian cancer is the most lethal gynecologic disease because usually, it is lately sensed, easily acquires chemoresistance, and has a high recurrence rate. Recent studies suggest that ovarian cancer stem cells (CSCs) are involved in these malignancies. Here, we demonstrated that galectin-3 maintains ovarian CSCs by activating the Notch1 intracellular domain (NICD1). The number and size of ovarian CSCs decreased in the absence of galectin-3, and overexpression of galectin-3 increased them. Overexpression of galectin-3 increased the resistance for cisplatin and paclitaxel-induced cell death. Silencing of galectin-3 decreased the migration and invasion of ovarian cancer cells, and overexpression of galectin-3 reversed these effects. The Notch signaling pathway was strongly activated by galectin-3 overexpression in A2780 cells. Silencing of galectin-3 reduced the levels of cleaved NICD1 and expression of the Notch target genes, Hes1 and Hey1. Overexpression of galectin-3 induced NICD1 cleavage and increased expression of Hes1 and Hey1. Moreover, overexpression of galectin-3 increased the nuclear translocation of NICD1. Interestingly, the carbohydrate recognition domain of galectin-3 interacted with NICD1. Overexpression of galectin-3 increased tumor burden in A2780 ovarian cancer xenografted mice. Increased expression of galectin-3 was detected in advanced stages, compared to stage 1 or 2 in ovarian cancer patients, suggesting that galectin-3 supports stemness of these cells. Based on these results, we suggest that targeting galectin-3 may be a potent approach for improving ovarian cancer therapy.

## INTRODUCTION

Ovarian cancer, the fifth leading cause of cancer-related death in women, is the most lethal disease among all gynecologic malignancies [1]. Ovarian cancer is a heterogeneous disease; however, epithelial ovarian carcinoma (EOC) is the major form of the disease and accounts for approximately 90% of ovarian tumors [2]. Subtypes of epithelial ovarian cancers can be divided into two groups: type I and type II [3]. Type 1 tumors are low-grade, slow growing, generally confined to the ovary at diagnosis, and develop from well-established precursor lesions; whereas, type 2 tumors are high-grade and rapidly progressing, for which well-defined precursor lesions have not been described [4]. Moreover, gene expression studies have shown that high-grade tumors cluster separately from low-grade and borderline tumors, suggesting that the two groups of tumors have a different genetic makeup [5, 6]. These data underscore the need for studies that probe the underlying molecular mechanism of ovarian cancer.

The combination of surgery and platinum-based chemotherapy was the standard treatment for advanced ovarian cancer [1]. Although a deeper understanding of disease progression has led to the development of numerous molecular targeting agents, recurrence still commonly occurs within 18 months of first line treatment in 70% of patients. The five-year survival rate of patients with advanced ovarian cancer is only 30.6% [7]. Recently, scientists have proposed that the cancer stem cells (CSCs) or cancer-initiating cells (CICs) are one of the reasons for disease relapse [4]. Traditional chemotherapy can kill the majority of cancer cells, while failing to target CSCs. Because CSCs has the properties as like normal stem cells, which are more resistant to DNA damage induced cell death than their more committed progeny, leading to a short-term survival advantage at the expense of the long-term maintenance of their genomic integrity [8]. It suggests that initial treatment increased the proportion of drug-resistant CSCs, resulting in recurrence of disease. Therefore, defining ovarian CSCs and identifying the regulatory molecules are a critical therapeutic approach for effective treatment.

Galectin-3 is a β-galactose-containing glycoconjugate-binding lectin. The amino acid sequence of galectin-3 contains a C-terminal carbohydrate recognition domain (CRD) that binds to β-galactosides, and an N-terminal domain with critical multivalent behavior [9]. The function of galectin-3 as an extracellular protein is well known, because of its ability to cross-link and cluster integrins; however, intracellular galectin-3 also functions as regulator of cellular processes [10]. Moreover, studies suggest galectin-3 regulates cancer progression, including cell proliferation, apoptosis, migration, and invasion [11–13]. In ovarian cancer, galectin-3 increases drug resistance and is also associated with poor survival rates in ovarian cancer patients [14–16]. These data strongly suggest that galectin-3 regulates stemness of ovarian cancer cells; however, additional studies are needed to elucidate the molecular mechanisms and phenotypes of cancer stem cells.

In this study, we determined the molecular mechanism by which galectin-3 increases the proliferation of ovarian cancer cells *in vivo* and *in vitro*. Moreover, we determined a role for galectin-3 in the maintenance of ovarian CSCs. Our results suggest that galectin-3 may be a potent target for ovarian cancer therapy.

## RESULTS

### Galectin-3 regulates cancersphere formation, which is a stem cell like-phenotype, during cultivation of ovarian cancer cells

We monitored galectin-3 expression in ten different ovarian cancer cell lines using RT-PCR and western blot analysis (Supplementary Figure S1A). We also determined the ability of cancersphere formation in ten ovarian cancer cells (Supplementary Figure 2). Among of them, A2780, OVCAR3, OVCAR429, SNU-251 and SKOV3 showed high ability for cancersphere formation. From these experiments, we selected A2780 and OVCAR3, as galectin-3 low-expressing cells, and SKOV3 and OVCAR429, as galectin-3 high-expressing cells, for further study.

**Figure 1:**
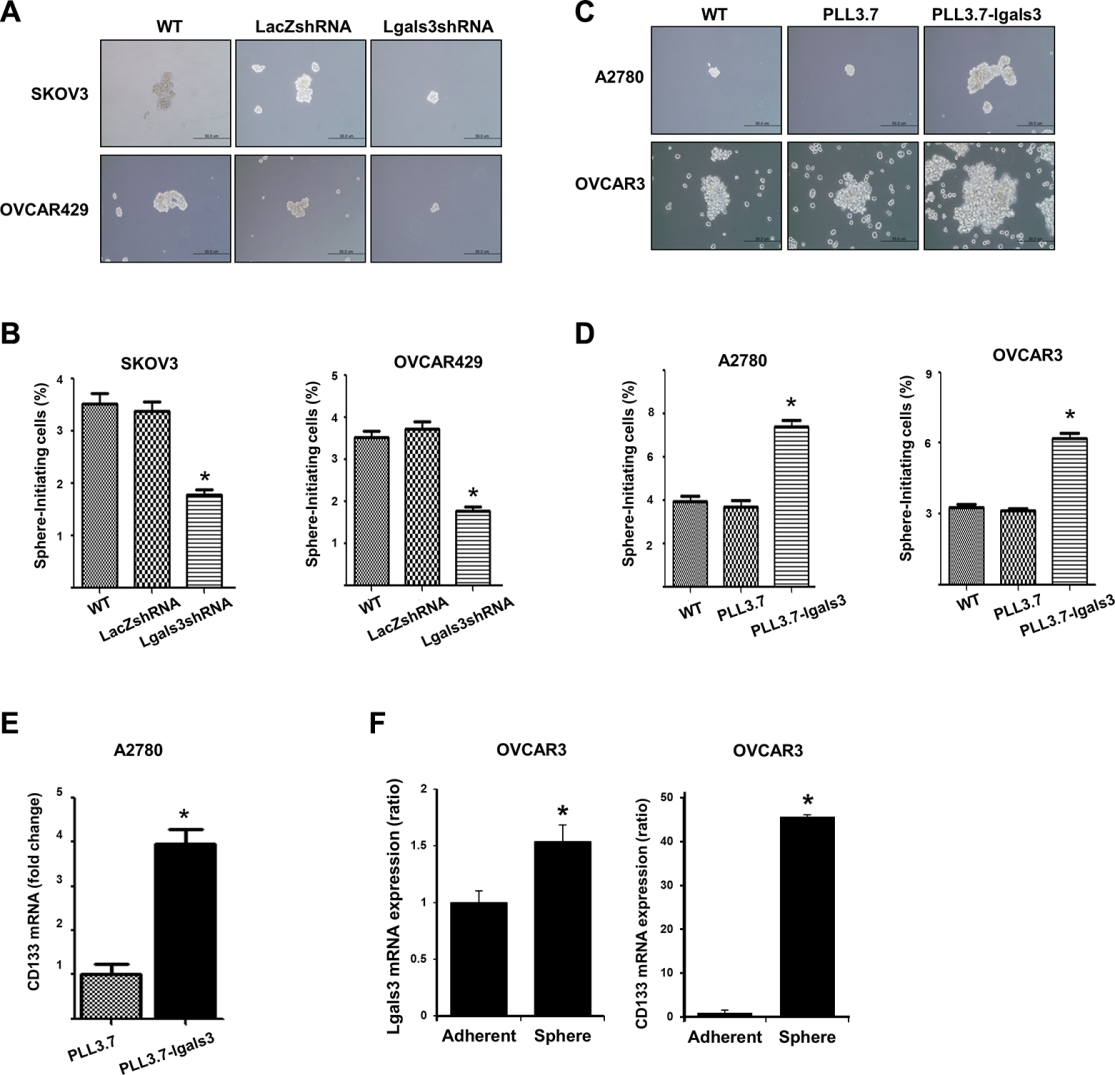
Galectin-3 regulates cancersphere formation, which is a stem cell like phenotype, in ovarian cancer cells (**A** and **B**) Detection of ovarian cancerspheres prepared by galectin-3-depleted SKOV3 and OVCRA429 cells or (**C** and **D**) galectin-3-overexpressed A2780 and OVCAR3 cells. (A and C) Galectin-3 shRNA containing lentiviruses (A) or galectin-3 overexpression vector containing lentiviruses (C) were infected in cells and stable galectin-3-depleted cells were selected by puromucin. These cells were cultured for 14 days and the morphology was taken by photographes. Scale bar represents 50 μm. (B and D) Galectin-3-depleted ovarian cancerspheres (B) or galectin-3-overexpressed (D) ovarian cancer spheres were collected and separated into single cells by trypsin treatment. The sphere forming cell numbers were calculated and prepared graphs by ratio of total seeded cell numbers. (**E**) Detection of CD133 expression level in galectin-3 overexpressed A2780 cells. The expression level was detected by real-time PCR analysis. (**F**) Detection of galectin-3 and CD133 expression level in OVCAR3 ovarian cancer spheres formed cells. The data are presented as the mean ± SD (*n* = 3). Significant differences are indicated by an asterisk (*p < 0.05), and the *p* values were calculated using the Student's *t* test.

To determine the effect of galectin-3 on the sphere formation of ovarian cancer cells, we prepared galectin-3-depleted cells by treating galectin-3 specific shRNA in galectin-3 high-expressing SKOV3 and OVCAR429 cells (Supplementary Figure S1B). After cultivation in sphere forming culture media, the sphere size and the number of spheres were both smaller in galectin-3 depleted cells than in control cells (Figure 1A) Total number of cells to form cancer sphere were also lower than control cells (Figure 1B and Supplementary Figure S3A).

We also prepared galectin-3-overexpressed cells by transforming PLL3.7-galectin-3 containing plasmids into the galectin-3 low-expressed A2780 and OVCAR3 cells (Supplementary Figure S1C). The sphere size and the number of spheres were larger for galectin-3-overexpresed cells than for the control cells (Figure 1C). Total number of cells to form cancer sphere were also more than control cells (Figure 1D and Supplementary Figure S3B).

The expression of the stem cell marker, CD133, also significantly increased in galectin-3-overexpressed A2780 cells (Figure 1E). Moreover, both CD133 and galectin-3 expression was increased after sphere forming cultivation of OVCAR3 cells (Figure 1F). These data suggest that galectin-3 increases cancer stem cell property in ovarian cancer cells.

### Galectin-3 regulates cell proliferation and chemotherapeutic agents-induced cell death in ovarian cancer cells

**Figure 2:**
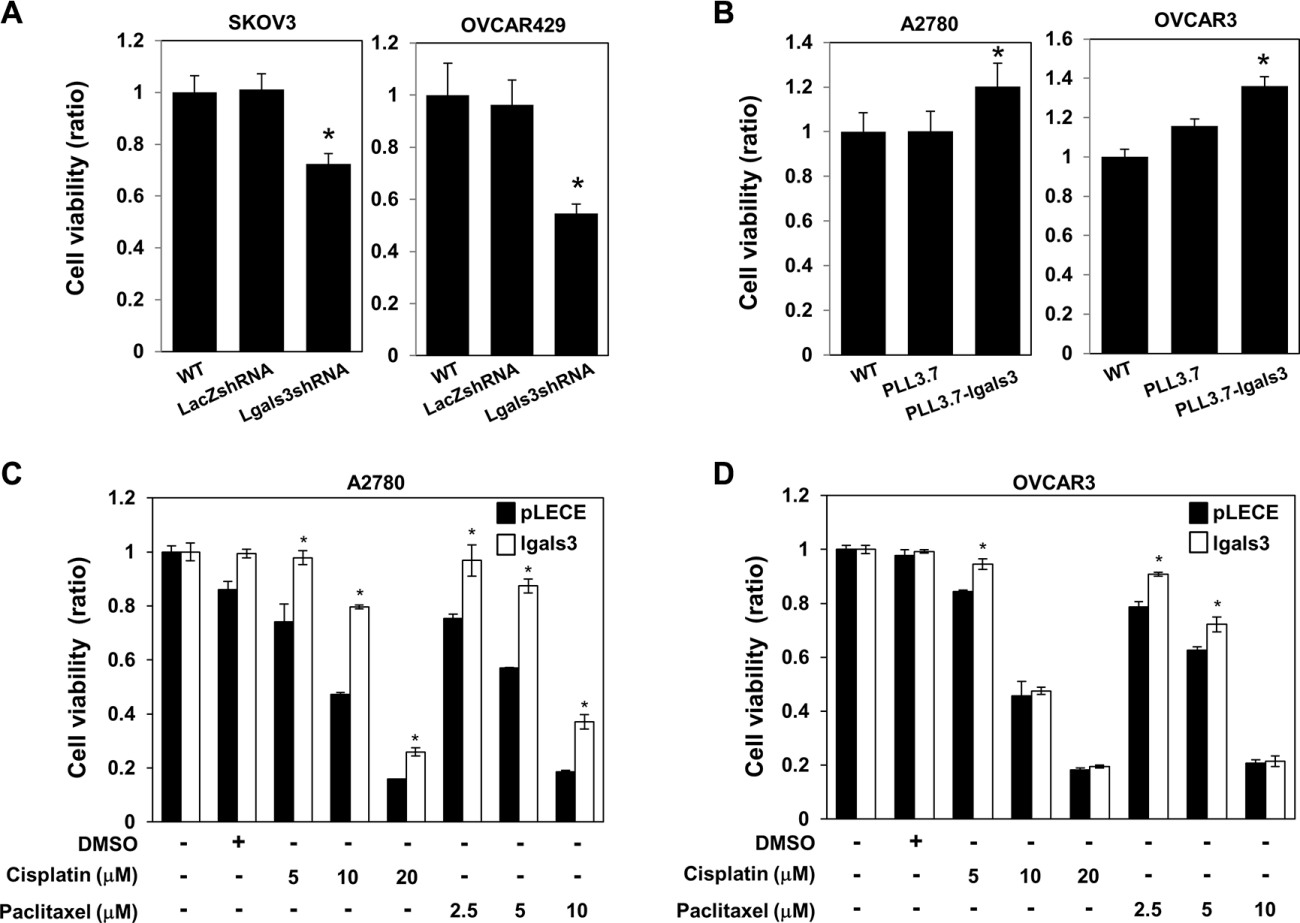
Galectin-3 regulates cell proliferation and drug resistance in ovarian cancer cells (**A** and **B**) (A) galectin-3 shRNA was transfected in SKOV3 cells and OVCAR429 cells, and (B) galectin-3 overexpression vector was transfected in A2780 cells and OVCAR3 cells. LacZ shRNA and PLL3.7 mock vector were used as the transfection control. Cell viability was analyzed by WST assays. (**C** and **D**) galectin-3 overexpression vector was transfected in A2780 cells and OVCAR3 cells. pLECE mock vector was used as a transfection control. After chemotherapeutic drugs, indicated paclitaxel, cisplatin, treatment for 48 hrs, cell viability was measured by WST assay. The data are presented as the mean ± SD (*n* = 3). Significant differences are indicated by an asterisk (*p < 0.05). The *p* values were calculated using the Student's *t* test.

Depletion of galectin-3 induced the cell proliferation in SKOV3 and OVCAR429 cells (Figure 2A) and overexpression of galectin-3 increased the cell proliferation in A2780 and OVCAR3 cells (Figure 2B). Interestingly, overexpression of galectin-3 significantly inhibited the cisplatin and paclitaxel-induced cell death of A2780 cells (Figure 2C) and OVCAR3 cells (Figure 2D). Moreover, depletion of galectin-3 enhanced paclitaxel-induced apoptosis in SKOV3 cells (Supplementary Figure S4A) and overexpression of galectin-3 reduced paclitaxel-induced apoptosis in A2780 cells (Supplementary Figure S4B). These data supposed that galectin-3 is involved in drug resistance, which is a phenotype of cancer stem cells, to protect the chemotherapeutic agents induced cell death.

### Galectin-3 regulates the invasion and migration of ovarian cancer cells

**Figure 3:**
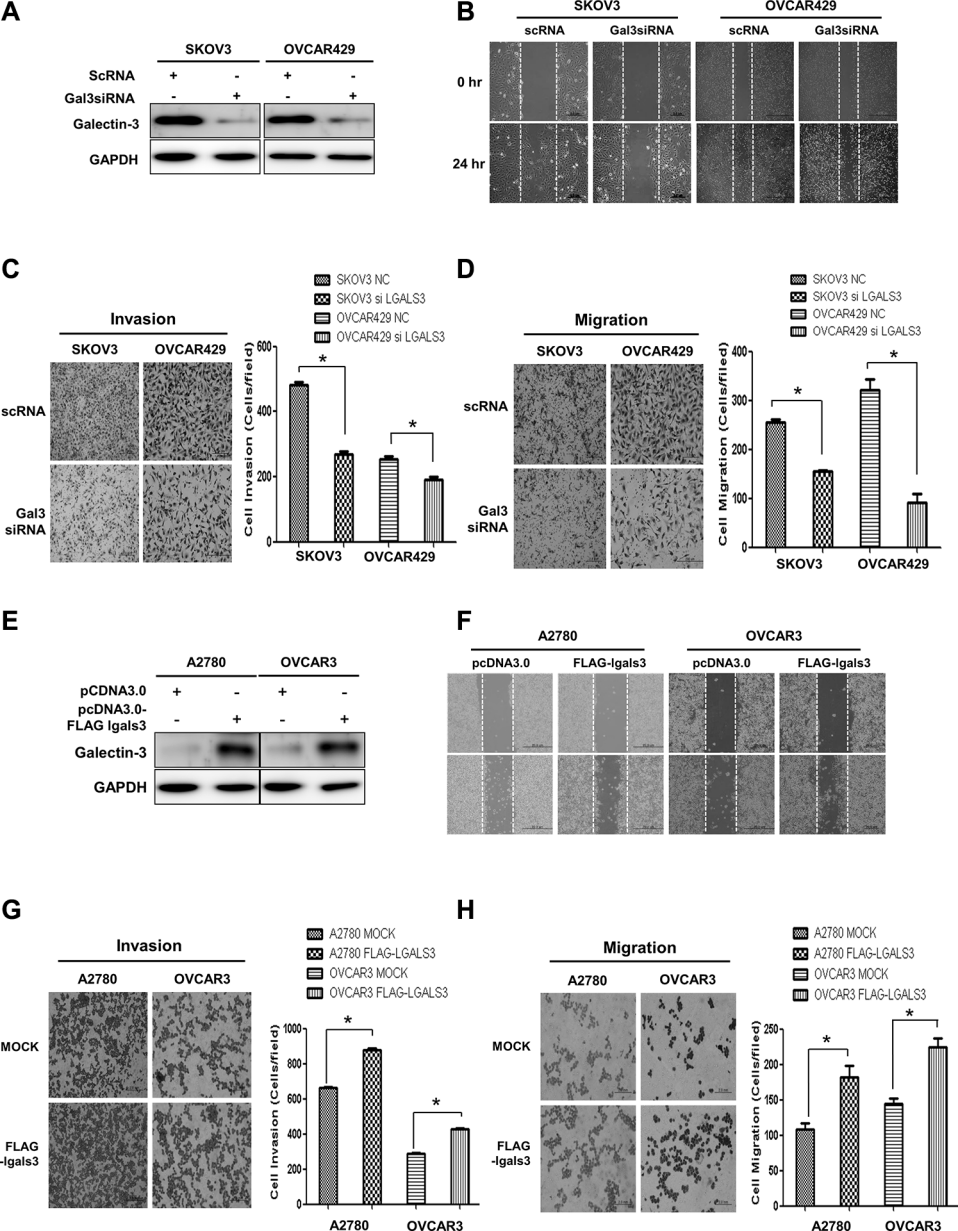
Galectin-3 regulates invasion and migration of ovarian cancer cells (**A**–**D**) SKOV3 cells and OVCAR429 were transfected with galectin-3 siRNA. Scrambled RNA (scRNA) was used as a transfection control. (A) Detection of galectin-3 protein expression by western blot analysis, (B) Detection of the healing ability by wound healing assays, (C) Invasion activity and (D) migration assay by trans-filter well assays. (**E**–**H**) A2780 and OVCAR3 cells was transfection with flag-tagged galectin-3 expression vector. pcDNA3.0 mock vector was used as a transfection control. (E) Detection of galectin-3 protein expression by western blot analysis, (F) Detection of the healing ability by wound healing assays, (G) Invasion activity and (H) migration assay by trans-filter well assays. Data are presented as mean ± SD (*n* = 3). The significant differences are indicated by asterisk (*p < 0.05), *p* values were calculated using the Student's *t* tests.

We prepared galectin-3-depleted cells by treating SKOV3 cells and OVCAR429 cells with galectin-3 specific siRNA (Figure 3A), and performed wound healing (Figure 3B), invasion (Figure 3C), and migration (Figure 3D) assays. The motility of galectin-3-depleted SKOV3 cells and OVCAR429 cells was significantly reduced in these assays. We also prepared galectin-3-overexpressed A2780 and OVCAR3 cells (Figure 3E) and performed wound healing (Figure 3F), invasion (Figure 3G), and migration (Figure 3H) assays. Overexpression of galectin-3 increased the motility of A2780 and OVCAR3 ovarian cancer cells. These results suggested that galectin-3 promotes the cell invasion and migration in ovarian cancer cells.

### Overexpression of galectin-3 increases the expression of genes involved in stemness regulating signaling pathways

**Figure 4:**
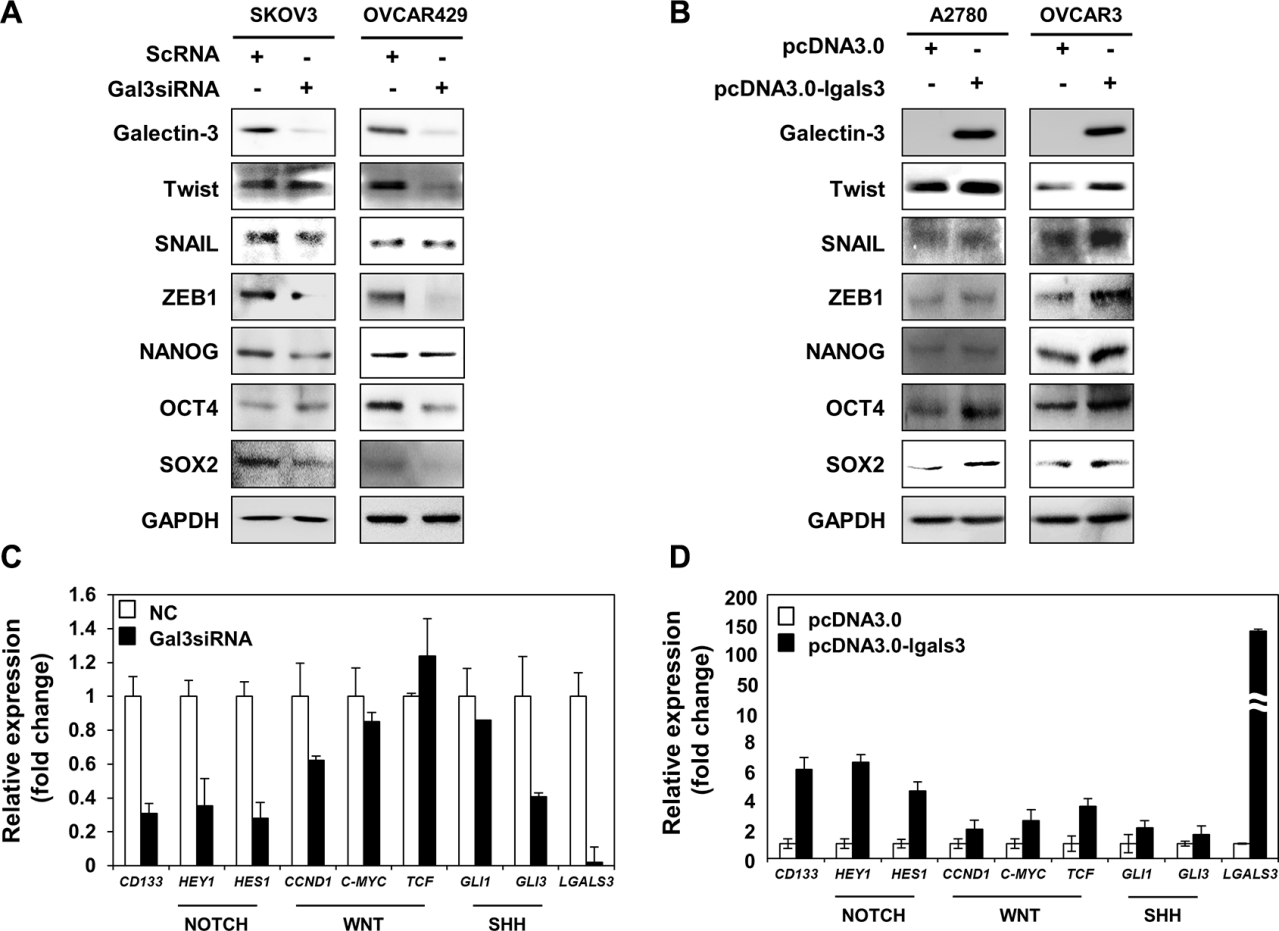
Galectin-3 regulates the Notch signaling and cancer stemness factors (**A** and **B**) Stemness and dedifferentiation related proteins were detected by western blot analysis in (A) galectin-3 siRNA transfected SKOV3 cells and OVCAR429 cells and (B) galectin-3 overexpressing vector transfected A2780 cells and OVCAR3 cells. Scrambled RNA (scRNA) and pcDNA3.0 mock vector were used as the transfection control, respectively. GAPDH was used as a loading control for western blot analysis. (**C** and **D**) (C) RT and real-time PCR analysis of *CD133*, *HEY1*, *HES1*, *CCND1*, *C-MYC*, *TCF*, *GLI1*, *GLI3*, and *LGALS3* abundance in scRNA or galectin-3 siRNA transfected SKOV3 cells. (D) Real-time PCR analysis of *CD133*, *HEY1*, *HES1*, *CCND1*, *C-MYC*, *TCF*, *GLI1*, *GLI3*, and *LGALS3* abundance in galectin-3-overexpressing vector transfected A2780 cells. Scrambled RNA (scRNA) and pcDNA3.0 mock vector were used as the transfection control, respectively. The expression level of GAPDH was analyzed for normalization control.

We determined the regulators of stemness phenotype, such as EMT regulators and stemness factors in absence of galectin-3 in SKOV3 and OVCAR429 cells (Figure 4A) and in overexpression of galectin-3 in A2780 and OVCAR3 cells (Figure 4B). After depletion of galectin-3, EMT regulators such as TWIST and SNAIL were slightly decreased and ZEB1 was significantly decreased in both SKOV3 and OVCAR429 cells. Stemness factors, such as NANOG, OCT4 and SOX2 were drastically decreased in galectin-3-depleted SKOV3 and OVCAR429 cells (Figure 4A). Moreover, we detected that EMT regulators, TWIST, SNAIL and ZEB1 were slightly increased and NANOG, OCT4 and SOX2, stemness factors were drastically increased in galectin-3-overexpressed A2780 and OVCAR3 cells (Figure 4B).

To determine how galectin-3 could regulate the stemness phenotype of ovarian cancer cells, we measured the mRNA expression of Notch, WNT/β-catenin, and SHH signaling pathway related genes in galectin-3-depleted SKOV3 cells (Figure 4C). These signaling pathways were previously reported to be involved in maintaining cancer stem cells. Interestingly, depletion of galectin-3 reduced the expression of most target genes for these pathways. Among them, the expression of the Notch target genes such as Hes1 and hey1 were strongly decreased by depletion of galectin-3 (Figure 4C). We also analyzed the mRNA expression levels of these genes in in galectin-3-overexpressing A2780 cells (Figure 4D). The expression of HES1 and HEY1 was significantly increased by galectin-3 overexpression. These results suggested that galectin-3 significantly regulates stemness factors through Notch signaling pathway in ovarian cancer cells.

### Galectin-3 induces cleavage and nuclear translocalization of Notch1 intracellular domain (NICD1) and the expression of the Notch target genes, such as HES1 and HEY1

**Figure 5:**
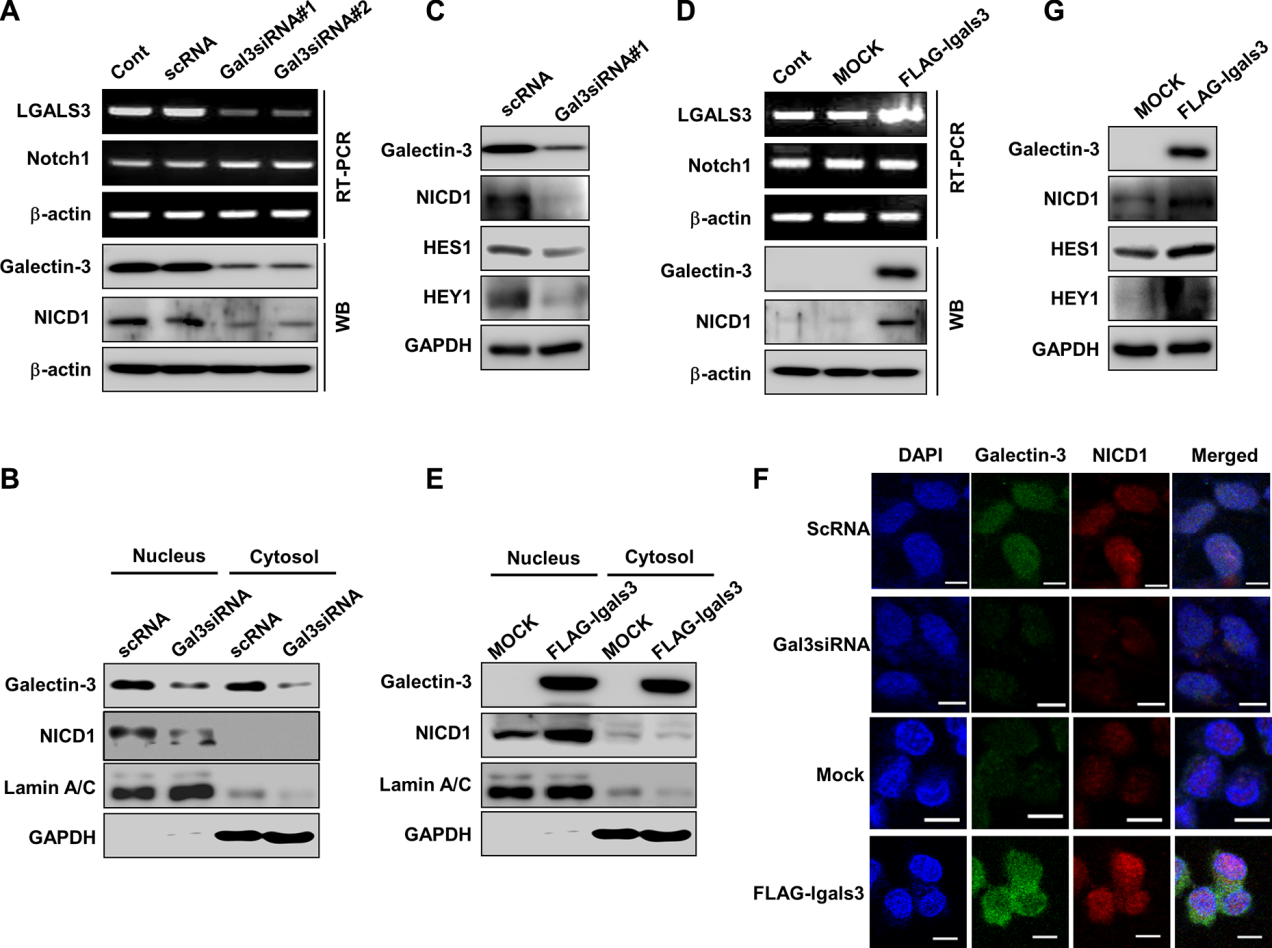
Galectin-3 induces the cleavage and nuclear translocation of Notch1 intracellular domain (NICD1) and the expression of the Notch target genes HES1 and HEY1 (**A**) Detection of mRNA and protein expression of galectin-3, Notch1 and NICD1 in galectin-3 silenced SKOV3 cells by RT-PCR and immunoblotting, respectively. β-actin was used as a loading control. (**B**) Protein levels of NICD1 and galectin-3 were detected in the nuclear and cytosol fractions of SKOV3 cells that were transfected with scRNA or galectin-3 siRNA. GAPDH and Lamin A/C were used as loading controls. (**C**) Protein levels of galectin-3, NICD1, HES1, and HEY1 in SKOV3 cells transfected with scRNA or galectin-3 siRNA. GAPDH was used as a loading control. (**D**) Detection of mRNA and protein expression of galectin-3, Notch1 and NICD1 in A2780 cells transfected with control vector or with the galectin-3 expression vector by RT-PCR and immunoblotting, respectively. β-actin was used as a loading control. (**E**) Protein levels of NICD1 and galectin-3 were detected in the nuclear and cytosol fractions of A2780 cells that were transfected with control or galectin-3 expression vector. GAPDH and Lamin A/C were used as loading controls. (**F**) Protein levels of galectin-3, NICD1, HES1, and HEY1 in A2780 cells transfected with mock vector or galectin-3 overexpressing vector. GAPDH was used as a loading control. (**G**) Immunocytochemical analysis was performed for detection of co-localization NICD1 and galectin-3 after transfection with scRNA or galectin-3 siRNA in SKOV3 cells (upper) and transfection with mock vector or galectin-3 expression vector (lower) in A2780 cells. Scale bar represents 50 μm. DAPI staining was used for detection of nucleus and the procedure was described in “Materials and Methods”.

We next determined how galectin-3 regulates Notch signaling in ovarian cancer cells. After silencing galectin-3 with two different galectin-3 specific siRNAs in SKOV3 cells, Notch1 expression did not change; however, levels of the cleaved form of the Notch1 intracellular domain (NICD1) decreased (Figure 5A). Silencing galectin-3 significantly reduced the levels of nuclear localized NICD1 (Figure 5B), and the expression levels of the Notch1 target genes, HES1 and HEY1 in SKOV3 cells (Figure 5C). Not only in SKOV3 cells, we also determined the effect of galectin-3 on NICD1 formation in SNU-840, DOV13 and RMUG-1 ovarian cancer cells (Supplementary Figure 5). Depletion of galectin-3 reduced the expression of cleaved NICD1 in these cells.

Galectin-3 overexpression did not affect the expression of the Notch1 genes, but induced the cleaved form of NICD1 (Figure 5D). We detected increased levels of NICD1 in nuclear fractions of galectin-3 overexpressing cells by western blot (Figure 5E). The expression of Hes1 and Hey1 also increased in galectin-3-overexpressing A2780 ovarian cancer cells (Figure 5F). Interestingly, we detected the colocalization of galectin-3 and NICD1 in nucleus of SKOV3 cells, whereas depletion of galectin-3 diminished their expression in nucleus (Figure 5G). Overexpression of galectin-3 induced accumulation of NICD1 in nucleus and colocalization of galectin-3 and NICD1 in A2780 cells (Figure 5G).

Interestingly, silencing of Notch1 with two different Notch1 specific siRNAs did not effect on the expression level or nuclear localization of galectin-3 in SKOV3 cells (Supplementary Figure S6A and S6B), and NCID1 overexpression did not induce the galectin-3 expression in A2780 cells (Supplementary Figure 6C). These data strongly suggest that overexpression of galectin-3 increases the cleavage and the nuclear translocalization of NICD1 in ovarian cancer cells.

### The CRD domain of Galectin-3 directly interacts with NICD1

**Figure 6:**
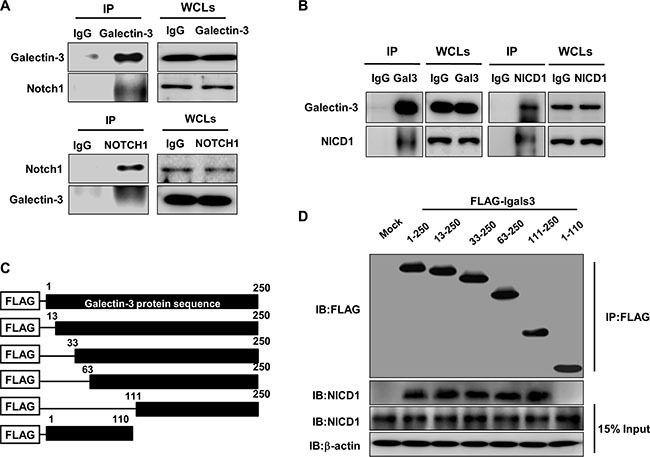
Galectin-3 interacts with NOTCH1 and NICD1 through its C-terminal domain (**A** and **B**) Immunoprecipitation was performed using (A) antibodies against galectin-3 and Notch1 or (B) antibodies against galectin-3 and NICD1 to detect interactions between galectin-3 and NICD1 in the SKOV3 cells. (**C**) Schematic model of Flag-galectin-3 domain (full length: amino acids 1–250, and CRD truncations: amino acids 33–250, 63–250, and 111–250). (**D**) Immunoprecipitation of Flag-galectin-3 domains and NICD1 using transfection of the galectin-3 domains in A2780 cells. β-actin was used as the loading control.

We tested the ability of galectin-3 to interact with Notch1 using an immunoprecipitation assay (IP) (Figure 6A). We detected interaction between galectin-3 and Notch1 in reciprocal IP assays using antibodies against galectin-3 and Notch 1, which suggests that galectin-3 directly interacts with Notch1. We then performed the IP assay using antibodies against cleaved NICD1 and galectin-3 (Figure 6B). These data suggested that galectin-3 interacted with the intracellular domain of Notch1.

Moreover, we prepared plasmids containing the galectin-3 peptide domain (Figure 6C). These plasmids were trasfected in A2780 cells. After performing IP assay using FLAG antibodies, we found that NICD1 interacted with amino acids 111–250, which is the carbohydrate recognition domain of galectin-3 (Figure 6D).

### Overexpression of galectin-3 increases the tumor growth in ovarian cancer cells xenografted mice

**Figure 7:**
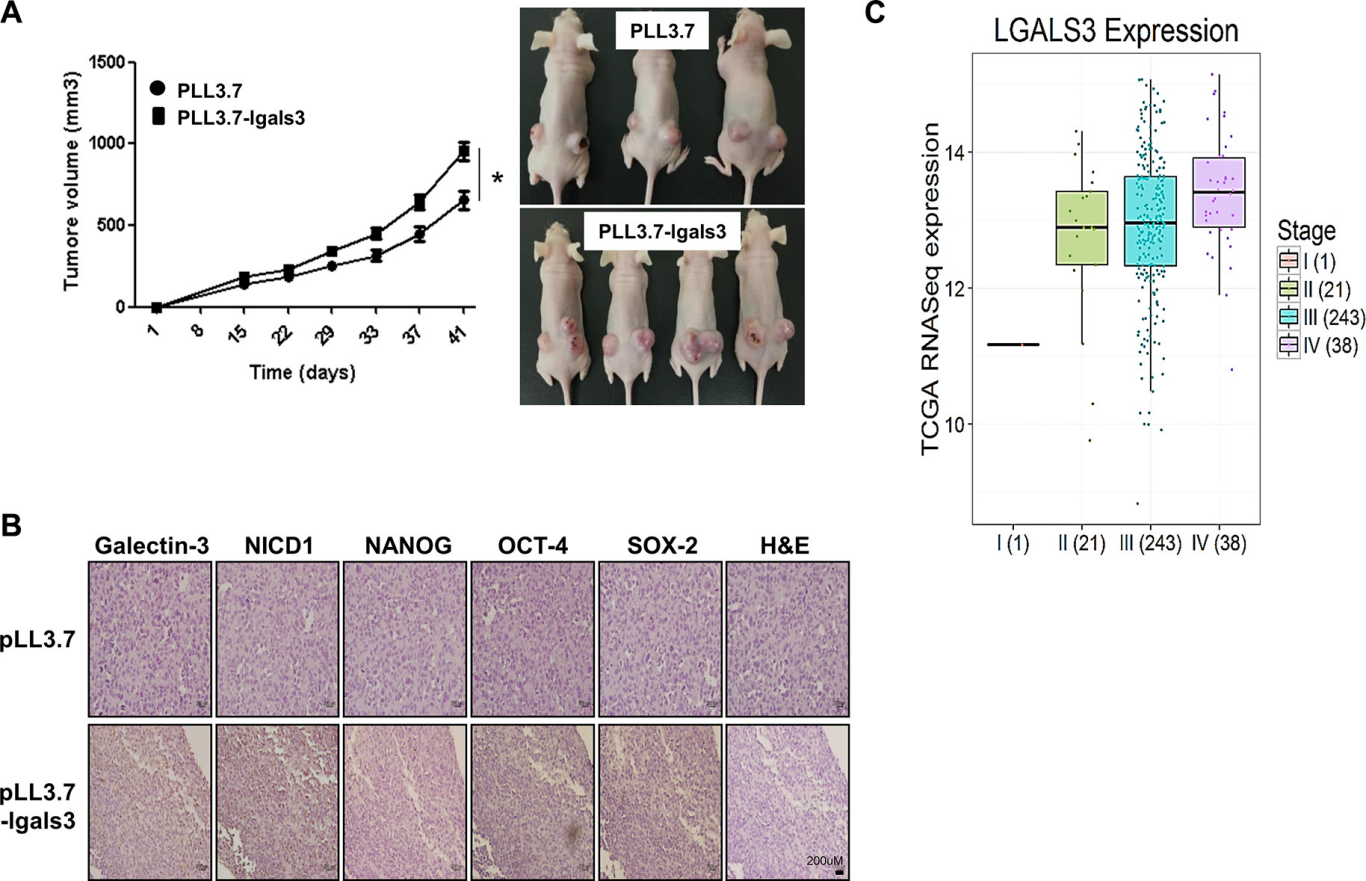
Overexpression of galectin-3 increases the tumor growth in xenografted mouse tumors A2780cells were transfected galectin-3 overexpression vectors and these cells were performed subcutaneous inoculation in nude mice. (**A**) Tumor formation was quantified by measuring the tumor volume 15 days after inoculation and represent as a graph (*n* = 3., left panel). The error bars indicate 95% confidence intervals; *p < 0.05 using two sided *t*-test. All statistical tests were two sided. Tumor formation was observed 41 days after inoculation and taken by photpgraphs (right panel) (**B**) Immunohistochemistry (IHC) analysis was performed to detect the expression level of galectin-3, NICD1, NANOG, OCT4, and SOX2 in tumors in *in vivo* mouse models. Method of IHC analysis was described in “Materials and Methods”. Scale bar presents 200 μm. (**C**) The expression level of galectin-3 was analyzed by stages in malignant tissues of ovarian cancer patients from TCGA database. The analysis method was described in “Materials and methods”.

We performed xenograft assays using galectin-3-overexpressing A2780 cells on nude mice (Figure 7A and 7B). The size of tumors prepared by galectin-3 overexpressing A2780 cells was larger than that of control A2780 cells. The growth rate of galectin-3 overexpressing A2780 cells were also faster than those of parent cells (Figure 7A). We determined the expression level of NICD1 and stemness factors, such as NANOG, OCT4 and SOX-2 in tumor sections prepared galectin-3 overexpressing A2780 cells (Figure 7B). Compared to tumor sections prepared by control A2780 cells, the expression of NICD1, NANOG, OCT4 and SOX2 was significantly increased in those prepared by galectin-3 overexpressing A2780 cells.

### Expression of galectin-3 is higher in malignant tissues of stage 4 ovarian cancer patients than in those of stage 2 ovarian cancer patients

We analyzed the expression level of galectin-3 in malignant tissues of ovarian cancer patients using TCGA public database (Figure 7C). The expression is increased according to stages; the expression of galectin-3 is higher in stage 4 than in stage 1. It strongly supports that galectin-3 promotes the malignancy of ovarian cancer.

## DISCUSSION

Cancer stem cells (CSCs), or cancer-initiating cells (CICs), have been increasingly studied over the past decade as a malignant and aggressive cancer phenotype. Although the origin of these cells is not fully understood, tumor heterogeneity, and the small populations of cells with stem-like characteristics, have been identified in many malignancies and are hypothesized to form the clonogenic core of tumor tissues [17, 18]. Such cells potentially originate from a more-differentiated cancer cell that acquires self-renewal properties, and the ability to undergo the epithelial-to-mesenchymal transition (EMT) [19]. Despite recent findings, the CSC hypothesis remains controversial. This controversy arises as a consequence of the technical and logistical challenges in isolating and identifying CSCs from human solid tumors that contain heterogeneous cell populations, and the limited number of validated surrogate assays currently available to substantively confirm stem-cell-like properties. However, surrogate assays for CSCs have been established that involve the formation of secondary ‘spheroids’ in suspension culture and the generation of 3D organoids [20].

Therefore, we examined the major properties of CSCs, spheroids formation and cell motility, which are the result of EMT. After depletion of galectin-3 expression, we performed suspension cultures for spheroids formation and drug sensitivity, and invasion and migration assays. Interestingly, depletion of galectin-3 significantly reduced spheroid formation, drug resistance and ovarian cancer cell motility. These results strongly suggested that galectin-3 increases the stem-like characteristics of ovarian cancer cells. Previously, it was reported that EGFR-dependent SOX2 expression or β-catenin activation by galectin-3 regulates lung cancer stem cells [21, 22]. We also reported that galectin-3 interacts with GSK-3b and then regulates WNT signaling to promote gastric cancer motility [9]. To identify the signaling pathways that are involved in galectin-3 dependent regulation of ovarian CSCs, we performed the depletion of galectin-3 in SKOV3 and overexpression of galectin-3 in A2780 cells and determined the signaling pathway related in CSC maintenance. CSCs display many features of embryonic or tissue stem cells, and typically demonstrate persistent activation of one or more highly conserved signal transduction pathways involved in development and tissue homeostasis, including the Notch, Hedgehog (HH), and WNT pathways [19]. Interestingly, we determined that Notch signaling related genes were drastically changed by galectin-3 expression. Depletion of galectin-3 significantly reduced the expression of HEY1 and HES1 in SKOV3 cells and overexpression of galectin-3 significantly increased their expression in A2780 cells, compared to target genes of the Hedgehog and WNT signaling pathways. Of course, overexpression of galectin-3 also increased target genes of Hedgehog and WNT signaling pathways, suggested that galectin-3 promotes ovarian CSCs by harmonized these signaling pathways, also. We are now studying how galectin-3 harmonized these signaling pathways.

We were interested that galectin-3 regulates the Notch1 signaling pathway in ovarian cancer stem cells because Notch pathway genes might be important therapeutic targets for CSCs and many new agents targeting the Notch pathways have entered clinical trials [23]. The Notch signaling pathway is an evolutionarily conserved pathway that plays an important role in development and cell fate determination, from angiogenesis to microenvironmental development, and immune response [23]. Notch is a large transmembrane protein that contains an EGF-like extracellular domain that is involved in ligand binding, a transmembrane domain, and an intracellular, nuclear localization domain. Ligand binding initiates cleavage of the intercellular domain, by the enzyme g-secretase. The cleaved domain, NCID, then translocates to the nucleus where it binds to the transcriptional complex, CBF-1 [24]. Previously, it was reported that OVCAR3, SKOV3, and CaOV3 cells, as well as a large portion (76%) of human ovarian papillary serous adenocarcinomas, express NICD, which is a potential target for new drug therapies in ovarian cancer [25]. Notch signaling is also a potential diagnostic marker of epithelial ovarian cancer [26]. Up-regulation of the Notch3 and increased expression of the Jagged2 was reported in ovarian cancers [27, 28]. Studies of the role of Notch1 in ovarian cancer indicated that mRNAs of the Notch pathway genes were highly expressed in ovarian adenocarcinomas, borderline tumors, and adenomas, and NCID1 overexpression increased cell proliferation and colony-formation capacity [29]. Sphere forming primary serous ovarian cancer cells also show increased resistance to cisplatin and paclitaxel [30]. These spheroid ovarian CSCs showed elevated levels of the CSC marker proteins CD44 and CD117, as well as increased Notch1 mRNA levels when compared to parental bulk tumor cells [31]. These previous reports suggest that cleaved NICD1 enhances stemness of ovarian CSCs. Here, we strongly suggest that galectin-3 is highly expressed and induces the cleavage and nuclear translocation of NICD1 in ovarian CSCs, which is an important regulatory mechanism of ovarian CSCs. Moreover, it is interesting that the carbohydrate-recognition domain (CRD) of galectin-3 interacted directly with NICD1. There are several hypotheses of why or how the galectin-3 CRD binds to NICD1 [32], and one possibility is that the galectin-3 CRD recognizes post-translationally glycosylated NICD1. We are currently testing this hypothesis with galectin-3.

Taken together, we conclude from this study that galectin-3 interacts with NICD1 and increases its cleavage and nuclear localization to support ovarian cancer stemness. Moreover, regulatory molecules for the Notch pathway may be critical therapeutic targets for blocking CSC self-renewal and proliferation, and tumor progression. Therefore, we strongly suggest that galectin-3 may be a potent target for regulating Notch1 signaling in ovarian cancer therapeutic strategies.

## MATERIALS AND METHODS

### Cell culture

The human ovarian cancer cell lines, A2780, DOV13, OVCAR3, OVCAR429, RMUG-I, RMUG-S, SKOV3, SNU-251, SNU-840, and TOV112D, were obtained from the Korea Cell Line Bank (KCLB, Seoul, Korea) and maintained in RPMI 1640, Ham's F-12 and Dulbecco's Modified Eagle Medium (DMEM) with 10% fetal bovine serum (FBS : Corning Costar, USA) and 1% antibiotics (Gibco, USA). Cells were cultured at 37°C in an atmosphere of 5% CO2.

### Mammosphere culture

Cells were grown in ultra-low attachment plates (Corning) and in Mammary Epithelium Basal Medium (MEBM; Lonza) supplemented with B27 (Gibco), 20 ng/mL of epidermal growth factor (EGF), and 20 ng/mL of fibroblast growth factor-basic (bFGF, PeproTech) at a density of 3,000 cells/mL as previously described [33]. After culturing the cells for 14 days, we counted mammospheres with diameters > 50 μm.

### Lentiviral vector construction

Lentiviral vectors for overexpression of galectin-3 were constructed by inserting the galectin-3 gene into the lentiviral vector pLL3.7 and pLECE3, as described previously [34]. Galectin-3 small hairpin RNA (shRNA)-lentiviral vectors were purchased from Sigma-Aldrich. Galectin-3 shRNA lentiviruses were prepared as described previously [35].

### siRNA transfection, Galectin-3 and NICD over-expression

Transfection with galectin-3, NICD expression vectors, as well as with galectin-3 and Notch1 siRNA, was performed using Lipofectamine 2000 and Lipofectamine RNAiMAX (Invitrogen), according to the manufacturer's instruction. Galectin-3 siRNA#1 (5′-AUAUGAAGCACUGGUGAGGUCUAUG-3′), galec tin-3 siRNA#2 (5′-GAAGAAAGACAGUCGGUUU-3′) and Notch1 siRNA#1 (5′-UCGCAUUGACCAUUCAAAC UGGUGGUU-3′), Notch1 siRNA#2 (5′-CGCUGCCUGG ACAAGAUCAAUU-3′) were purchased from Genolution (korea).

### Transwell migration and invasion assays

OVCAR429 and SKOV3 cells were transfected with scRNA and galectin-3 siRNA. A2780 and OVCAR3 were transfected with a control vector and galectin-3 expression vector. After transfection for 24 hrs, cells (A2780 – 1 × 10^4^, OVCAR3 – 1 × 10^4^, OVCAR429 – 1 × 10^4^, SKOV3 – 1 × 10^4^ in each well) were isolated and added to the upper Transwell (Corning Costar, USA) chambers with 0.5 mg/mL collagen type I (BD Bioscience, Korea)-coated filters for the migration assay, and with a 1/14 dilution of Matrigel (BD Bioscience, Korea)-coated filters for the invasion assays. RPMI 1640 (A2780, OVCAR3, SKOV3) or DMEM (OVCAR429) containing 10% FBS and 1% antibiotics was added to the lower chamber and incubation was continued for 24 hrs. Cells that migrated or invaded the lower chamber were quantified after H&E staining, as previously described [36]. For quantification, cells were counted in four randomly selected areas in each well using wide-field microscopy. Graphs were expressed as mean ± SD from three independent experiments.

### Total RNA isolation and reverse transcriptase polymerase chain reaction (RT-PCR)

Total RNA was extracted using TRIzol Reagent (Invitrogen) according to the manufacturer's instructions. Subsequently, RT-PCR, using the Reverse Transcription System (Promega, Madison, WI, USA), was performed as previously described [12]. The following primers were used: Lgals3, forward 5′-ATGGCAGACAATTT TTCGCTCC-3′ and reverse 5′-ATGTCACCAGAAATTCC CAGTT-3′; β-actin, forward 5′-AGCCTCGCCTTTGC CGA-3′ and reverse 5′-CTGGTGCCTGGGGCG-3′; Notch1, forward 5′- GAGGCGTGGCAGACTATGC-3′ and reverse 5′-CTTGTACTCCGTCAGCGTGA-3′; and GAPDH, forward 5′-GGCTGCTTTTAACTCTGGTA-3′ and reverse 5′-ACTTGATTTTGGAGGGATCT-3′. PCR was performed using Ex-Taq (TaKaRa, Shiga, Japan) according to the manufacturer's instructions [37].

### Quantitative RT–PCR

Total tissue RNA was prepared using TRIzol reagent (Invitrogen) according to the manufacturer's instructions. cDNA (3 μg) was synthesized from RNA using the Quantitative RT-PCR Master Mix (TOYOBO). The following primers were used: GLI1, forward 5′-TGGAT ATGATGGTTGGCAAGTG-3′ and reverse 5′-ACAGAC TCAGGCTCAGGCTTCT-3′; GLI3 forward 5′-GAAGTG CTCCACTCGAACAGA-3′ and reverse 5′-GTGGCTG CATAGTGATTGCG-3′; TCF1, forward 5′-CACGGGCA AACACTACGGT-3′ and reverse 5′-TTGACCTTCGAGT GCTGATCC-3′; C-MYC, forward 5′-TTCGGGTAGTGG AAAACCAG -3′ and reverse 5′-CAGCAGCTCGAATTTC TTCC -3′; CCND1, forward 5′-AACTACCTGGACCGC TTCCT-3′ and reverse 5′-CCACTTGAGCTTGTTCAC CA-3′; HES1, forward 5′-ATGGAGAAAAATTCCTCGTC CC-3′ and reverse 5′-TTCAGAGCATCCAAAATCAG TGT-3′; HEY1, forward 5′-GAAACTTGAGTTCGGCTC TAGG-3′ and reverse 5′-GCTTAGCAGATCCTTGCT CCAT-3′; and CD133, forward 5′- ACCAACACCAAGAA CAAGGC-3′ and reverse 5′-GGAGCTGACTTGAATT GAGG-3′. Real-time RT-PCR was performed using SYBR Green Master Mix (TOYOBO) with an ABI instrument (Applied Biosystems Inc.). The normalization control was B-actin.

### Western blot analysis and immunoprecipitation

Cell lysate extractions were prepared with RIPA buffer 1 % NP-40; 0.1% sodium dodecyl sulfate; 0.5% deoxycholate; 150 mM NaCl; 50 mM Tris, pH 7.5) and a protease inhibiter cocktail. 10–20 μg total protein of each lysate was resolved in SDS PAGE gels and electrotransferred to PVDF membranes, and then blocked in 5% skim milk in 0.05% Tween-20 with 1× PBS (PBST). Primary antibodies were incubated with the blots at a 1:1000 dilution in minimal volumes of 5% BSA (Bovine serum albumin) in PBST buffer for 1 hr at room temperature or over-night at 4°C. Anti-mouse or anti-rabbit goat-HRP-conjugated secondary antibodies were incubated at a 1:3000 dilution in 5% BSA in PBST buffer for 1 hr at room temperature. Antibodies used in this study were anti-galectin-3, anti-GAPDH, anti-Lamin A/C, anti-Notch1, anti-ZEB1, anti-SNAIL, anti-TWIST, anti-Nanog, anti-Sox2, and anti-Oct4 that were purchased from Santa Cruz Biotechnology. Anti-activated Notch1 (NICD1) was obtained from Abcam. Anti-c-MYC and anti-HES1 were purchased from Cell Signaling. Membranes that were probed with primary antibodies and secondary antibodies were detected by ECL solution (Amersham Life Science) using a LAS-3000 (Fujifilm) detector, according to the manufacturer's directions. Immunoprecipitation was carried out using A/G agarose beads coated with anti-galectin-3 (Santa Cruz Biotechnology), anti-FLAG (Sigma) and anti-activated Notch1 (Cambridge, MA, USA). The proteins were detected by western blot analysis using antibodies against anti-galectin-3, anti-activated Notch1, and anti-Notch1. Mouse/rabbit IgG (Santa Cruz Biotechnology) was used as a negative control [38].

### Fractionation of cellular extracts

Nuclear and cytoplasmic extracts were prepared from SKOV3 and A2780 cells after treatment with galectin-3 siRNA and transfection with galectin-3 overexpression vectors. Cells were lysed in Buffer A (10 mM HEPES (pH 7.9), 1.5 mM Mgcl2, 10 mM KCl, 1 mM DTT, 0.2 mM PMSF, 0.1% NP-40) containing a Xpert protease inhibitor cocktail (GenDEPOT, Barker, TX, USA) and phosphatase inhibitor (NaF, Na3VO4), incubation on ice for 15 min. The cell lysate was centrifuged for 10 min at 850 G at 4°C and discard the supernatant. Next, resuspend the pellet with Buffer C (20 mM HEPES (pH 7.9), 25 % Glycerol, 0.42 M Nacl, 0.2 mM EDTA, 1.5 mM Mgcl2, 1 mM DTT, 0.2 mM PMSF) and vortex for 15 sec. Incubate the cell lysate for 30 min on ice and vortex every 10 min for 15 sec. After incubation the cell lysate were centrifuged for 10 min at 13,200 rpm at 4°C and collect the supernatant (Nuclear). Western blot analysis was performed as described previously [39] with nuclear fraction protein samples.

### Apoptosis detection assays

SKOV3 and A2780 cells were plated onto culture plates. Next day, cells were transfected with Galectin-3 siRNA or Galectin-3 expression vector. After 6 hrs, media is changed. And then treat with paclitaxel (5 μM) for 48 hrs. After the time passed, cells were harvested. Cells were washed twice with cold PBS and then resuspended in 1× Annexin V Binding at a concentration of 1 × 10^6^ cells/ml. Then, 100 μl of the solution (1 × 10^5^ cells) was transferred to a 1 ml culture tube and 5 μl of PE Annexin V and 5 μl 7-AAD each sample. The cells were gently vortexed and incubated for 15 min at RT in the dark. We added 400 μl of 1× Annexin Binding Buffer to each tube and transferred the solution to FACS filter tubes. Apoptosis distribution was measured by Annexin V staining using FACS LSRII.

### Cell viability analysis

Stable cells were plated in 96-well culture plates (5,000 cells/well). After 48 hrs, highly sensitive water-soluble tetrazolium salt (WST) solution (Daeil, Seoul, Korea) was subsequently added to each well. WST solution works through the cleavage of tetrazolium salt into a water-soluble formazan by the succinate-tetrazolium reductase system of the mitochondrial respiratory chain. It is active only in viable cells. After 1 hr of additional incubation, the plate was shaken gently. The absorbance was measured using an ELISA reader at a wavelength of 450 nm. Inhibition of cell proliferation by drugs (paclitaxel, cisplatin) was measured using the WST assay.

### Immunocytochemistry and immunohistochemistry

Immunocytochemistry was performed as described previously [9]. Briefly, cells in chamber slides were fixed with 4% formaldehyde at 4°C for 30 min, washed with PBS, and permeabilized in 0.5% Triton X-100 for 10 min. Cells were incubated with primary antibodies (galectin-3 and NICD) at 4°C and then were incubated with fluorescein isothiocyanate anti-mouse and Cy5 anti-rabbit secondary antibodies (Invitrogen), as well as with 4,6-diamino-2-phenylindole staining solution. Images were analyzed by confocal microscopy (LSM 700, Carl Zeiss). Immunohistochemistry for galectin-3, NICD, Sox-2, Oct-4, and Nanog of xenograft mouse tumor tissues was performed according to instructions, using a Vectastain ABC kit and DAB substrate kit (Vector Laboratories, Burlingame, CA).

### Animal experiments

All animal experiments were approved by the Institutional Review Board of the Yonsei University College of Medicine and were performed in specific pathogen-free facilities in accordance with the University's Guidelines for the Care and Use of Laboratory Animals (2014–0027). The preparation of xenografted mice was performed as described previously [40].

### Statistical analysis

Two tumors per mouse were obtained and the mean tumor volume per mouse was analyzed. Unpaired *t-test*s were used to analyze the mean tumor volume in xenografted mice. All statistical tests are two sided, and the values are expressed as the mean with 95% confidence intervals (CI). *P-value* < 0.05 was considered statistically significant. Statistical analyses were performed using GraphPad Prism (version 6; GraphPad Software Inc., La Jolla, CA, USA).

## SUPPLEMENTARY MATERIALS FIGURES


