# Supplementary material for: Galectin-3 supports stemness in ovarian cancer stem cells by activation of the Notch1 intracellular domain
Source: Oncotarget. 2016 Sep 9;7(42):68229–41. doi: 10.18632/oncotarget.11920 (PMC5356551; doi:10.18632/oncotarget.11920)
Supplement: Supplementary file 1 [file oncotarget-07-68229-s001.pdf]

Galectin-3 supports stemness in ovarian cancer stem cells by activation of the Notch1 intracellular domain

Supplementary Materials

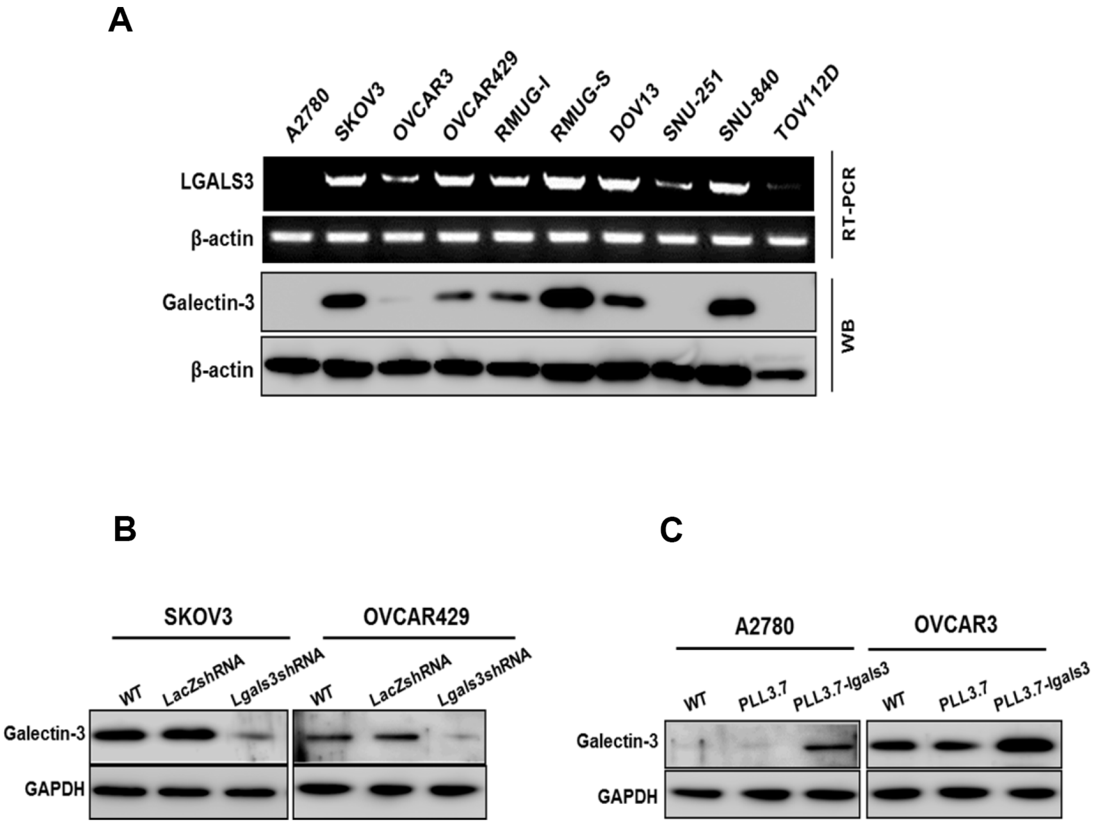

Supplementary Figure S1: Galectin-3 expression in ovarian cancer cell lines and galectin-3 depletion and overexpression in ovarian cancer cells. (**A**) Galectin-3 expression was detected by RT-PCR and western blot analysis in ovarian cancer cells. β-actin was used as a loading control. (**B**) Galectin-3 specific shRNA was transfected in SKOV3 cells and OVCRA429 cells. LacZshRNA was used as a transfection control. (**C**) Galectin-3 overexpressing vector was transfected in A2780 cells and OVCRA3 cells. PLL3.7 mock vector was used as a transfection control. Galectin-3 protein levels were detected by western blot analysis. GAPDH was used as a loading control.

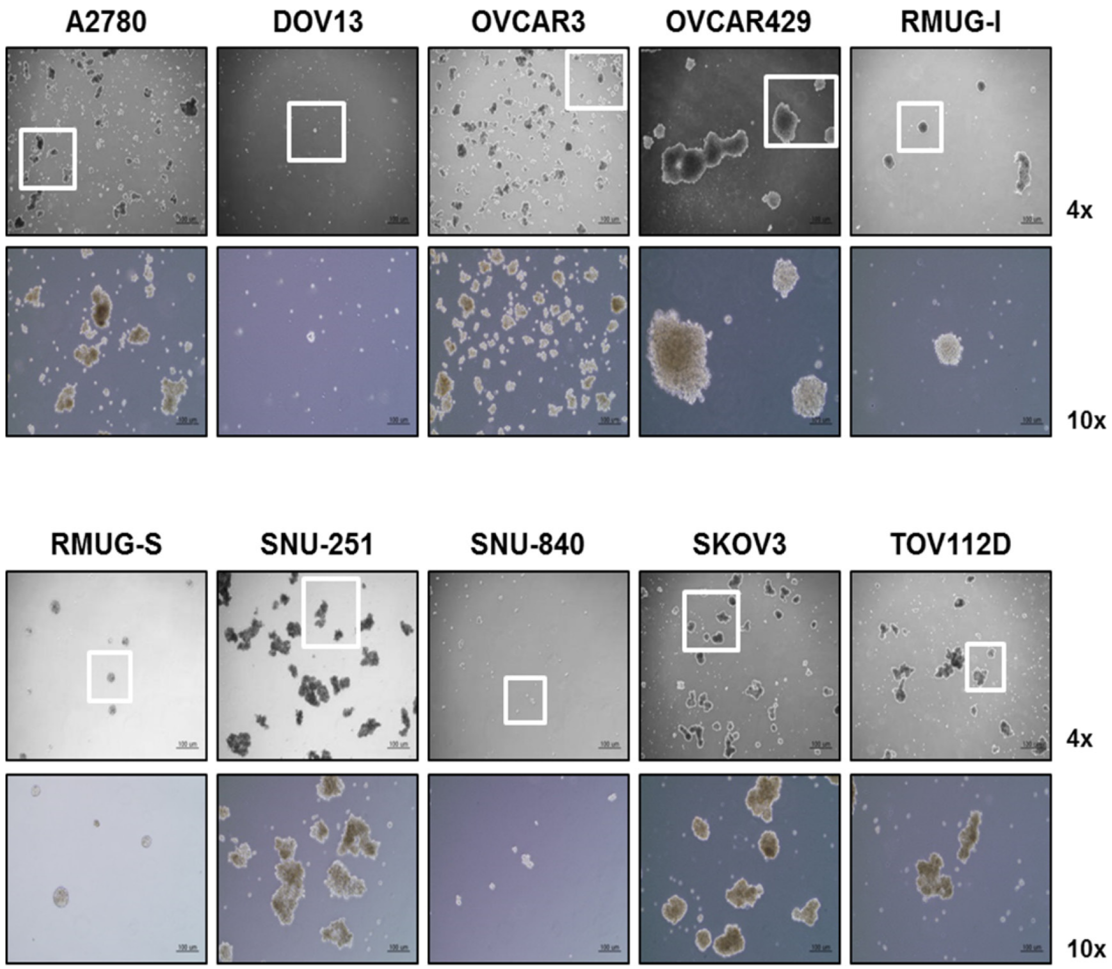

Supplementary Figure S2: Detection of cancersphere formation of ten ovarian cancer cells. The cancersphere forming ability was measured under sphere forming conditions for 14 days as described in "Materials Methods". Scale bar represents 100 μm. All experiments were performed independent triplicate in the indicated ovary cell lines.

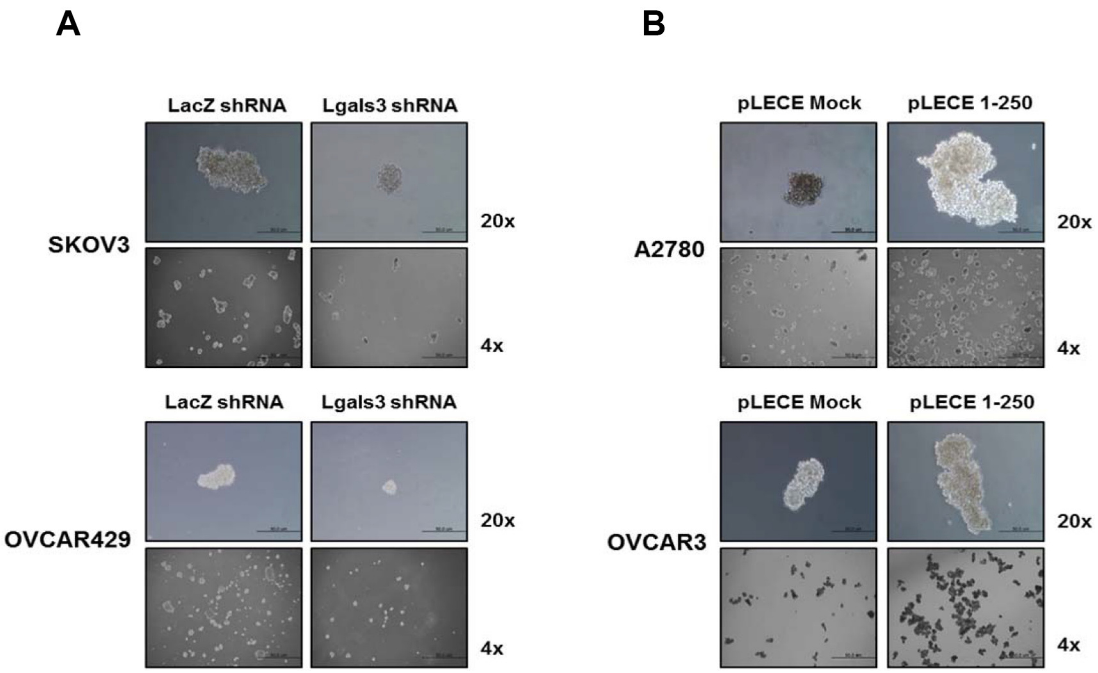

Supplementary Figure S3: Galectin-3 regulate cancersphere formation in ovary cancer cell lines. (**A**) Detection of cancerspheres prepared by galectin-3-silenced SKOV3 and OVCRA429 cells. (**B**) Detection of cancerspheres prepared by galectin-3-overexpressed A2780 and OVCRA3 cells. The cancerspheres forming ability was measured under sphere forming conditions for 14 days as described in "Materials Methods". Scale bar represents 50 µm. All experiments were performed independent triplicate in the indicated ovary cell lines.

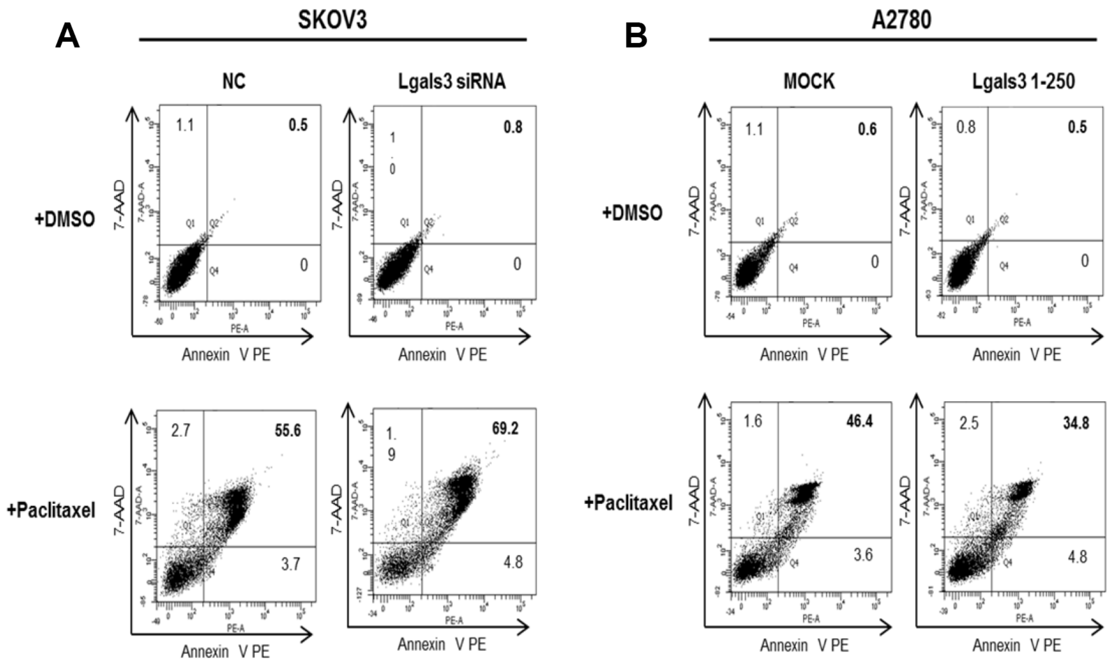

Supplementary Figure S4: Galectin-3 regulates induction of apoptosis in paclitaxel treated SKOV3 and A2780 ovary cancer cells. (**A**) Galectin-3 specific siRNA was transfected in SKOV3 cells. Scambled RNA (NC) was used as a transfection control. Apoptosis induction was measured by annexin-V staining analysis in galectin-3-silenced SKOV3 and (**B**) Galectin-3 overexpressing vector was transfected in A2780 cells. PLL3.7 mock vector was used as a transfection control. Galectin-3-overexpressed A2780 cells were treated paclitaxel (5 μM) treatment for 48 hrs. Apoptosis induction was described in “Materials and method” session and quantitative data are presented graphically.

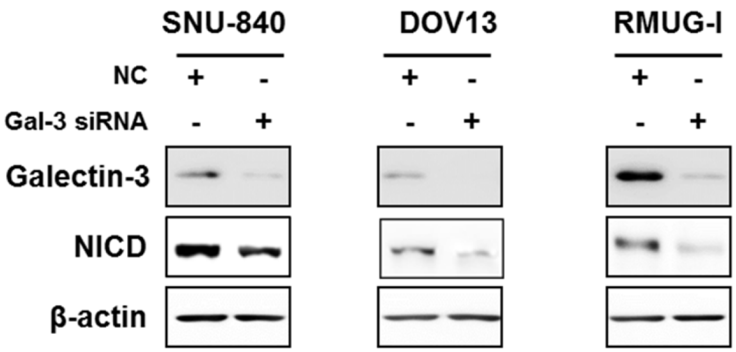

Supplementary Figure S5: Galectin-3 regulates Notch intracellular domain (NICD) formation in SNU-840, DOV13 and RMUG-I ovarian cancer cells. Detection of galectin-3 and NICD protein expression by western blotting in galectin-3-silenced SNU-840, DOV13, and RMUG-I cells. β-actin was used as a loading control.

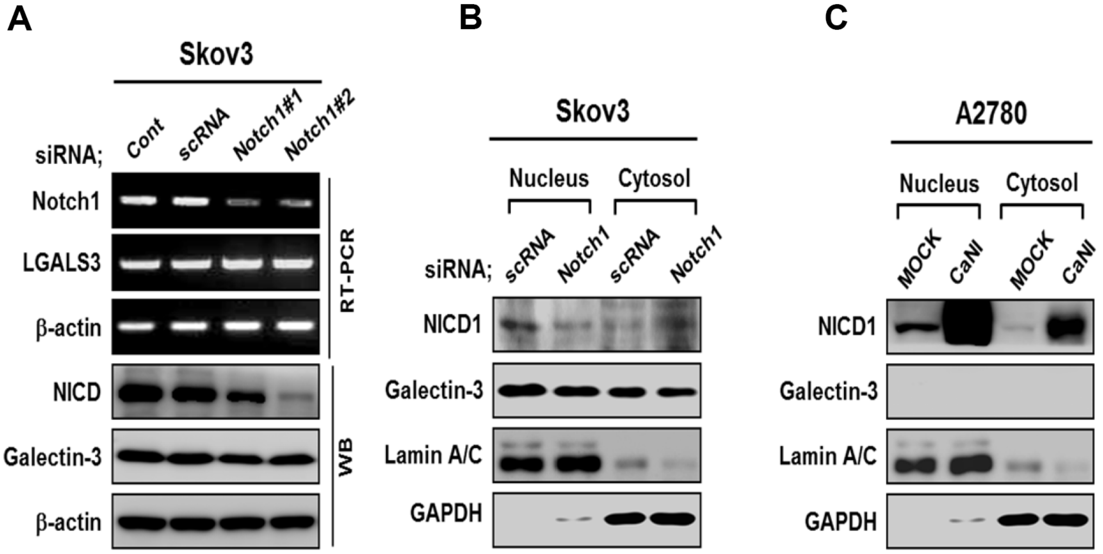

Supplementary Figure S6: Expression of Notch1 did not effect on the expression of galectin-3 in Skov3 and A2780 cells. (**A**) Expression of Nocth1 and galectin-3 were detected by RT-PCR and NICD and galectin-3 were detected by RT-PCR western blot analysis in SKOV3 cells that were transfected with scRNA or Notch1 siRNA. β-actin was used as a loading control. (**B**) Protein levels of NICD1 and galectin-3 were detected in the nuclear and cytosol fractions of SKOV3 cells that were transfected with scRNA or Notch1 siRNA. GAPDH and Lamin A/C were used as loading controls. (**C**) Protein levels of NICD1 and galectin-3 were detected in the nuclear and cytosol fractions of A2780 cells that were transfected with pcDNA4.0 CaNI (NICD1 overexpression). GAPDH and Lamin A/C were used as loading controls.
